# Supplementary material for: PepVAE: Variational Autoencoder Framework for Antimicrobial Peptide Generation and Activity Prediction
Source: Front Microbiol. 2021 Sep 30;12:725727. doi: 10.3389/fmicb.2021.725727 (PMC8515052; doi:10.3389/fmicb.2021.725727)
Supplement: Supplementary file 1 [file Data_Sheet_1.PDF]

Supplementary Material

1 Supplementary methods

Peptide characterization

The characteristics of sequences in the starting dataset and generated AMPs, including peptide length, amino acid composition, net charge, hydrophobicity, and hydrophobic moment, were assessed using the Python library modlAMP (Müller, Gabernet et al. 2017).

Preliminary methods comparisons

Machine learning methods were implemented using the MATLAB Statistics and Machine Learning Toolbox, and the Regression Learner app. The models tested included: Rational Quadratic Gaussian Process Regression, Fine Gaussian Support Vector Machine, Ensembles of Trees – Bagged, and Regression Trees – Coarse, as well as others part of the app. The Regression Learner app was used to select the most promising methods for further study. The use of structured data was necessary due to the default behavior of MATLAB that treats an array of character (char) data as a single string. The amino acid identities as the standard single-letter abbreviation were held in a table consisting of unit cell arrays which each contained a single char datum. To this was appended the response variable, the logarithm of the Minimum Inhibitory Concentration, log(MIC).

The amino acid sequences were of variable lengths. Since machine learning methods typically require equal length data, it is necessary to pad the data, either at the C-terminus, or by a multiple alignment method. C-terminal padding simply involves the addition of a non-coding character to the end of shorter peptide sequences to bring them all up to the length of the longest example. Multiple alignment was performed with the MATLAB function multialign from the Bioinformatics Toolbox. MATLAB machine learning treats the amino acid identities (as well as noncoding characters) as categorical type data and gives no special meaning to the actual amino acid.

Latent space analysis

As previously used to evaluate VAE-arrived clustering, methods described by Lopez *et al.* were used, with modification (Lopez, Regier et al. 2018). The 2D projections resulting from the dimensionality reduction methods PCA, t-SNE, and UMAP from the VAE-produced latent representation was used as input to the *K*-means algorithm and measure the overlap between the resulting clustering annotations and the pre-specified subpopulations (the < 0.2 log μM and >2 log μM labels) using Adjusted Rand index and Adjusted Mutual Information (AMI) measurements as metrics.

Minimum inhibitory concentration (MIC) assay analysis

Statistical analysis of predicted and experimental MIC data was performed using receiver operating characteristic (ROC) curve analysis and area under the curve (AUC) calculation from the pROC package (Robin, Turck et al. 2011) following categorization of predicted and experimental MICs into either >128 μM or ≤ 128 μM.

Circular dichroism analysis

Estimates of secondary structure from circular dichroism data. Measured ellipticity (mdeg) converted to $\Delta\varepsilon$ was input into the circular dichroism data analysis program Beta Structure Selection (http://bestsel.elte.hu) from which helix, antiparallel, parallel, turn, and other were obtained (Micsonai, Wien et al. 2018). Following summation of antiparallel, parallel, and turn into Sheet, the results were plotted for both groups A and B.

2 Supplementary Figures and Tables

2.1 Supplementary tables

Table S1. *E. coli* AMP and MIC values dataset used for training the VAE.

Peptide ID	Sequence	MIC (log µM)
0	A A A A A A A A A A G I G K F L H S A K K F G K A F V G E I M N S	2.09995035
1	A A A A A A A I K M L M D L V N E R I M A L N K K A K K	1
2	A A A K A A L N A V L V G A N A	1.903089987
3	A A G M G F F G A R	1.108873537
4	A A G R Y Q L L S R Y W D A Y R	2.176091259
5	A A H H I A R P I V H V G K T I H R L V T G	1.204119983
6	A A K H A A H R A	2.729534801
7	A A L R G A L R A V A R V G K A I L P H V A I A N P Y V R T P Y V H N N P	-0.096910013
8	A A N F G P S V F T P E V H E T W Q K F L N V V V A A L G K Q Y H	0.503730045
9	A A T G T G K T A A F A L P V L E R L I	2.10713582
10	A A Y L L A K I N L K A L A A L A K K I L	1.010088196
11	A D A D D D D D K	2.929418926
12	A E V A P A P A A A A P A K A P K K K A A A K P K K A G P S	0.301029996
13	A F F A R L L A S V R A A V K A F A K K P R L I G L S T L L	1.791834707
14	A F G M A L K L L K K V L	0.672097858
15	A F G V L A K V A A H V V P A I A E H F	1.806179974
16	A F H H I F R G I V H V G K T I H R L V T G	0.541853992
17	A F R K Q L K W	0.580520991
18	A G K K T I R Q Y L K N K I K K K W R K A V I A W	0.903089987
19	A G L Q F P V G R I G R L L R K	0.799340549

Table S2. Characteristics of the peptides in the study.

Peptide name	Group Id	Sequence	MW	Length	Hydrophobicity	Charge	Hydrophobic moment	pI	Boman index
p1	A	VLNANLLR	912.1	8	0.7	1.0	0.3	10.5	0.9
p2	A	VLIKTRLFIKRK	1515.0	12	0.2	5.0	0.3	12.5	1.9
p3	A	LNWKAILKHIIK	1476.9	12	0.3	3.1	0.6	11.1	-0.1
p4	A	VLPKVMAHMK	1153.5	10	0.5	2.1	0.4	10.8	-0.4
p5	A	LNWGAVLKHVVK	1363.7	12	0.5	2.1	0.5	10.8	-0.4
p6	A	LILKRKRKRRILI	1834.4	14	-0.6	8.0	0.3	13.0	3.7
p7	A	LNWGAIKKHIIK	1420.8	12	-0.1	3.1	0.4	11.1	0.3
p8	A	VLNENLLA	885.0	8	0.9	-1.0	0.1	3.8	-0.1
p9	A	LNWGAFLKHFFK	1507.8	12	0.2	2.1	0.5	10.8	-0.1
p10	A	VLNENLLH	951.1	8	0.2	-0.9	0.2	5.4	0.7
p11	A	VLNENAAR	886.0	8	-0.4	0.0	0.4	6.4	2.8
p12	A	VLNENLRR	1013.2	8	-1.0	1.0	0.7	10.4	4.5
p13	A	VLNENLLR	970.1	8	0.1	0.0	0.5	6.4	2.0
p14	A	VDLKNLLK	942.2	8	0.1	1.0	0.5	9.5	1.0
p15	A	VALNENLLR	1041.2	9	0.3	0.0	0.4	6.4	1.6
p16	A	LRRLRLRLLRLLRRLLRLL	2512.3	19	0.3	8.0	1.2	13.3	3.4
p17	A	VLNNLLR	841.0	7	0.6	1.0	0.9	10.5	1.3
p18	A	VLNENLAA	842.9	8	0.6	-1.0	0.2	3.8	0.3
p19	A	VLNEALLR	927.1	8	0.7	0.0	0.4	6.4	1.0
p20	A	LNWGAWLKHWWK	1624.9	12	-0.8	2.1	0.5	10.8	0.0
p21	A	LVKRVKKVL	1082.4	9	0.4	4.0	0.8	11.9	1.1
p22	A	VNLKNLLR	969.2	8	0.0	2.0	0.6	11.7	1.9
p23	B	KWKLWKKIEKWGQGIGAVLKWLTTWL	3197.9	26	-0.3	5.0	0.8	11.1	0.0
p24	B	KWKSFLKTFKSPVKTVFYTALKPISS	3032.7	26	-0.1	6.0	0.6	11.1	0.7
p25	B	KWKSFIKKLTSVLKKVVTTAKPLISS	2931.6	26	0.1	7.0	0.7	11.6	0.6
p26	B	KWKSFIKKLTSAAKKVVTTAKPLISS	2861.5	26	-0.1	7.0	0.7	11.6	0.8
p27	B	KWKSFLKTFKSPARTVLHTALKPISS	2972.6	26	-0.3	6.1	0.7	12.1	1.3
p28	B	KWKSFIKKLTSAAKKVLTTGLPALIS	2830.5	26	0.2	6.0	0.7	11.5	0.2
p29	B	KWKSFLKKLTSAAKKVLTTALKPISS	2875.5	26	-0.1	7.0	0.7	11.6	0.8
p30	B	KWKSFLKTFKSAVKTVLHTALKAISS	2920.5	26	0.1	6.1	0.7	11.5	0.7
p31	B	FIGGLRRLFATVVGTVVGAINKLGGG	2573.1	26	1.0	3.0	0.6	12.5	-0.4
p32	B	KFFKKLKKAVKKGFKKFAKV	2399.1	20	-0.6	10.0	0.9	11.8	1.3
p33	B	FFFHIIKGLFHAGRMIHGLV	2340.9	20	1.1	2.3	0.7	11.7	-0.7
p34	B	FFFKLLPKAIGALKKI	1834.4	16	0.9	4.0	0.6	11.3	-1.0
p35	B	FKIKASKKLLKKVGKGALGAVAKALAQQA	2966.7	29	0.1	8.0	0.7	11.6	0.3
p36	B	KWKKFIKKLTSAAKKVLTTGLPALIS	2871.6	26	0.1	7.0	0.7	11.6	0.3
p37	B	KWKKFLKKLTSAAKKVLTTALKPISS	2916.6	26	-0.2	8.0	0.7	11.6	0.9
p38	B	FFKKFIGGVAKIAGKAAPHGVGQLIPHVTP	3086.7	30	0.4	4.2	0.7	11.3	-0.4

2.2 Supplementary figures

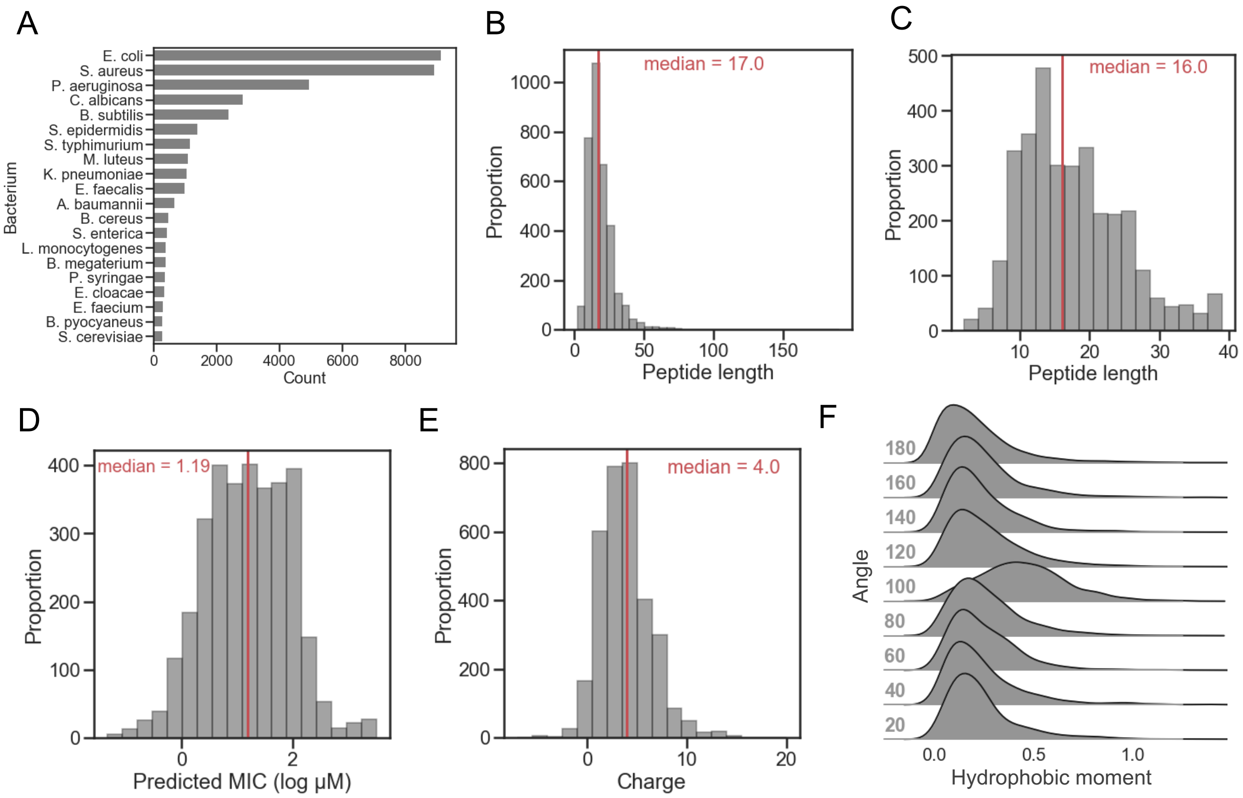

Figure S1. **Characteristics of the AMP dataset**. A) Top 20 bacteria tested against ordered by count in the initial GRAMPA dataset. *E. coli*, *S. aureus,* and *P. aeruginosa* are most commonly tested, with *E. coli* the most counted at 9150. B) Peptide length distribution of AMPs (without cysteine) tested against *E. coli*. C) Peptide length distribution of AMPs (without cysteine) tested against *E. coli*, with AMPs of length ≥ 40 removed. D, E, and F show the distribution of MIC (log μM), charge, and hydrophobic moments at different angles, respectively, of the filtered peptides represented in panel C. Medians for distributions shown in B, C, D, and E are indicated with a red line.

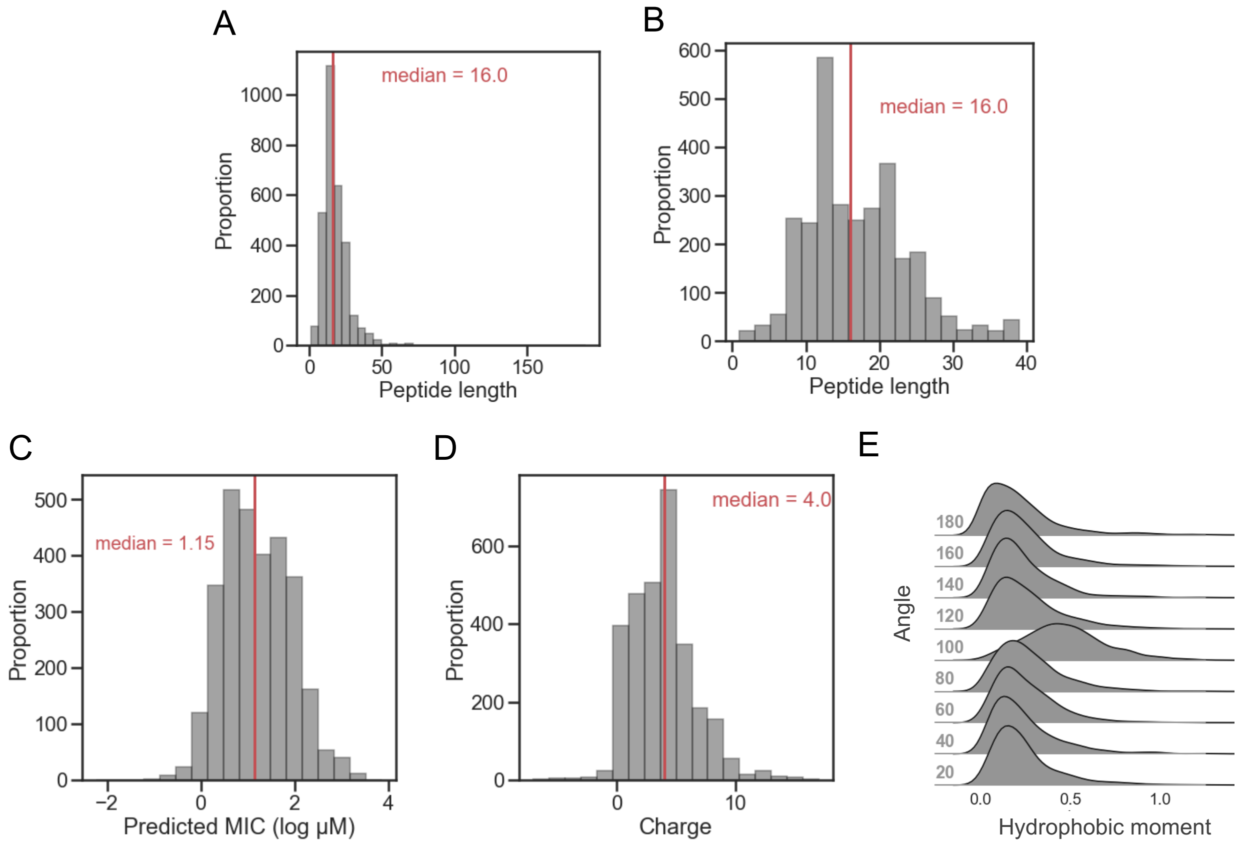

Figure S2. **Characteristics of the AMP dataset for *S. aureus*.** A) Peptide length distribution of AMPs (without cysteine) tested against *S. aureus*. B) Peptide length distribution of AMPs (without cysteine) tested against *S. aureus*, with AMPs of length ≥ 40 removed. C, D, and E show the distribution of MIC (log µM), charge, and hydrophobic moments at different angles, respectively, of the filtered peptides represented in panel C. Medians for distributions shown in A, B, C, and D are indicated with a red line.

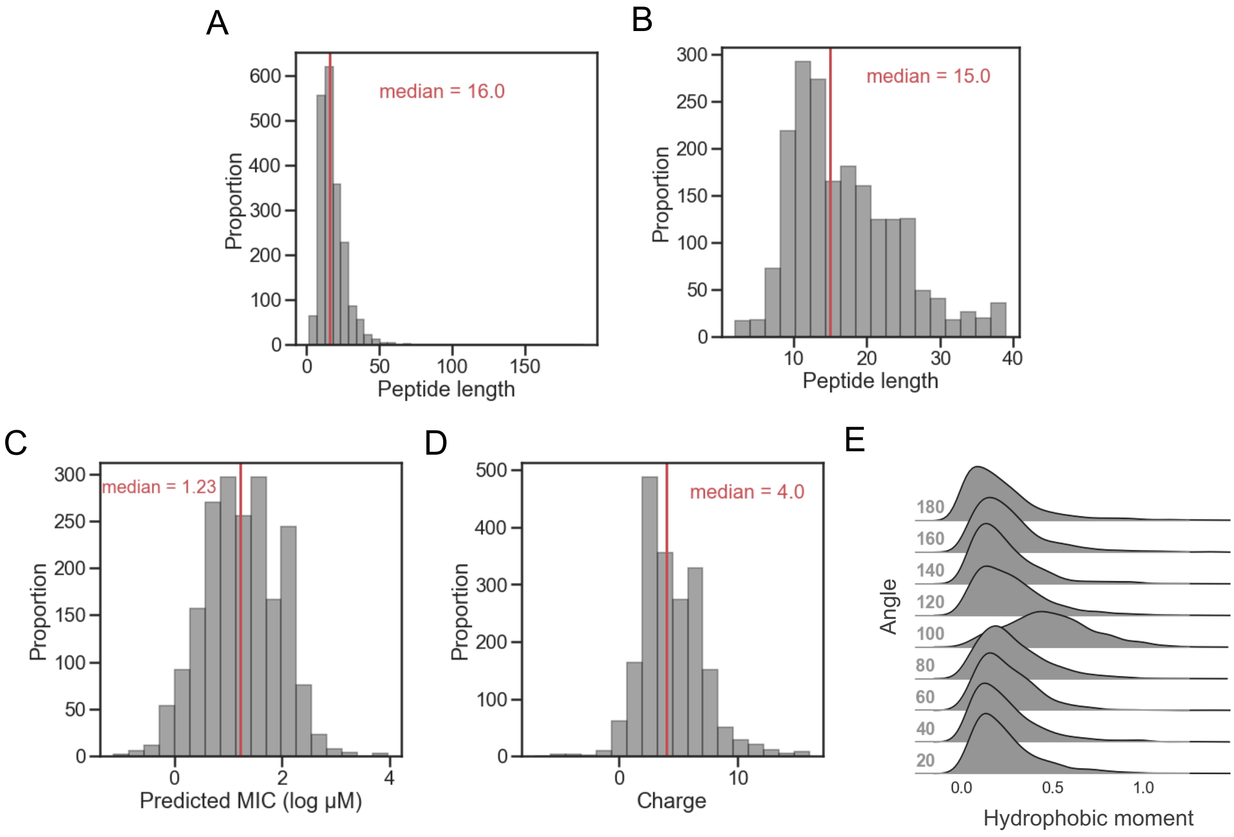

Figure S3. **Characteristics of the AMP dataset for *P. aeruginosa*.** A) Peptide length distribution of AMPs (without cysteine) tested against *P. aeruginosa*. B) Peptide length distribution of AMPs (without cysteine) tested against *P. aeruginosa*, with AMPs of length ≥ 40 removed. C, D, and E show the distribution of MIC (log µM), charge, and hydrophobic moments at different angles, respectively, of the filtered peptides represented in panel C. Medians for distributions shown in A, B, C, and D are indicated with a red line.

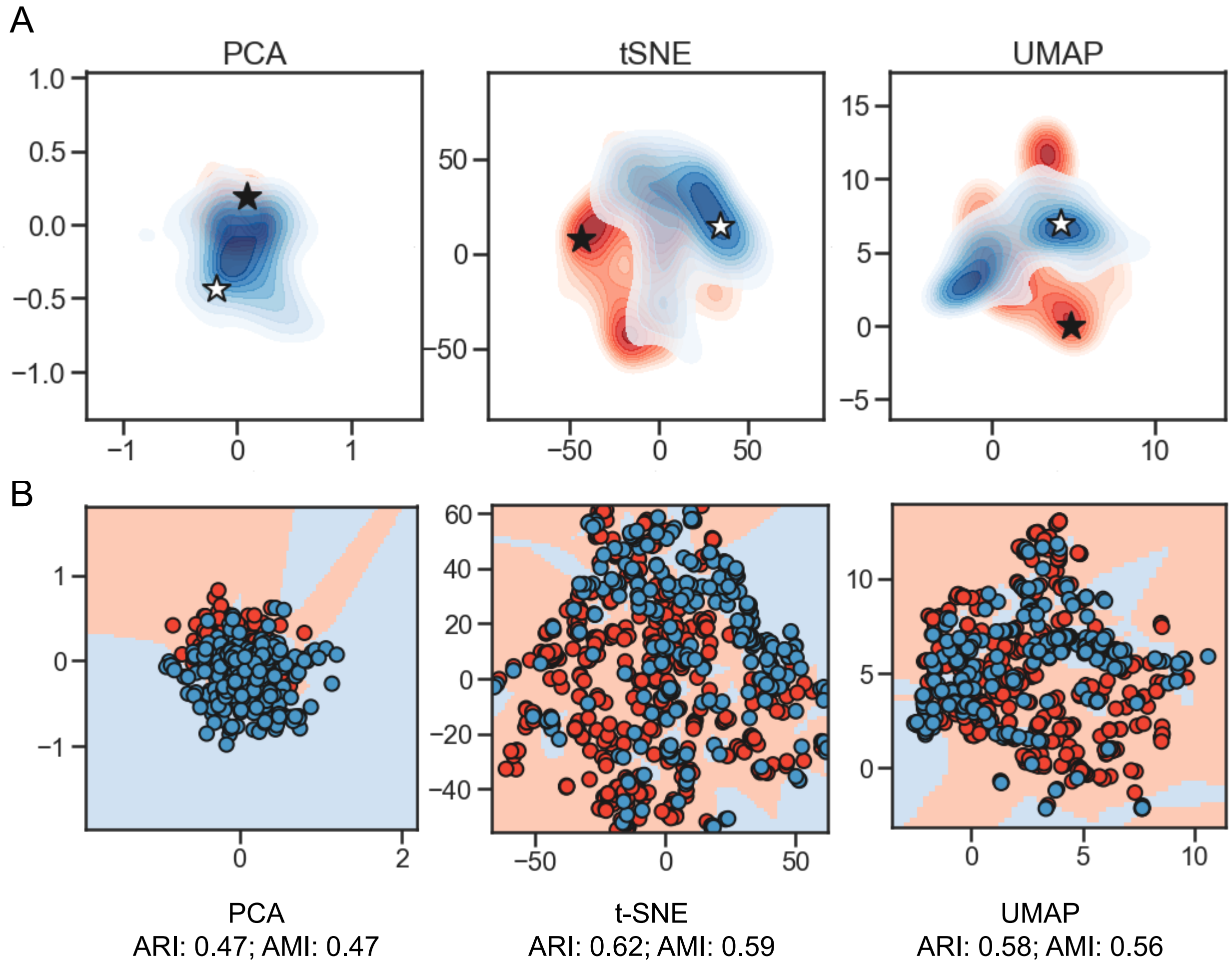

Figure S4. **Dimensionality reduction for visualization of the 50-dimension latent space.** A) Three different techniques were used: PCA, t-SNE, and UMAP (each with two components). PCA and t-SNE were imported from Scikit-learn; UMAP was from McInnes *et al.* (McInnes, Healy et al. 2018). The MIC thresholds for coloring were: < 0.2 log µM is shown in blue, >2 log µM was set to red, and those with values ≥ 0.2 and ≤ 2 were set to light gray. For t-SNE, perplexity set to 30, and learning rate set to 100. UMAP was performed using Bray-Curtis Similarity as the metric, with default settings. The black star is located at the peptide VLNENLLA, and a white star is located at the embedding most distant in cosine similarity (encoding for the peptide KFGKIVGKVLKQLKKVSAVAKVAMKKG). B) Adjusted Rand index (ARI) and Adjusted Mutual Information (AMI) measurements (for both, higher is better). KNN predictions performed on the 2D projections resulting from the dimensionality reduction methods PCA, t-SNE, and UMAP shown in (A), compared with the true labels were used to calculate the Rand index values shown above each plot. Scatterplot points represent locations of encoded peptides, background coloring indicates location of the KNN decision boundary. MIC thresholds for coloring: < 0.2 log µM is shown in blue, >2 log µM in red.

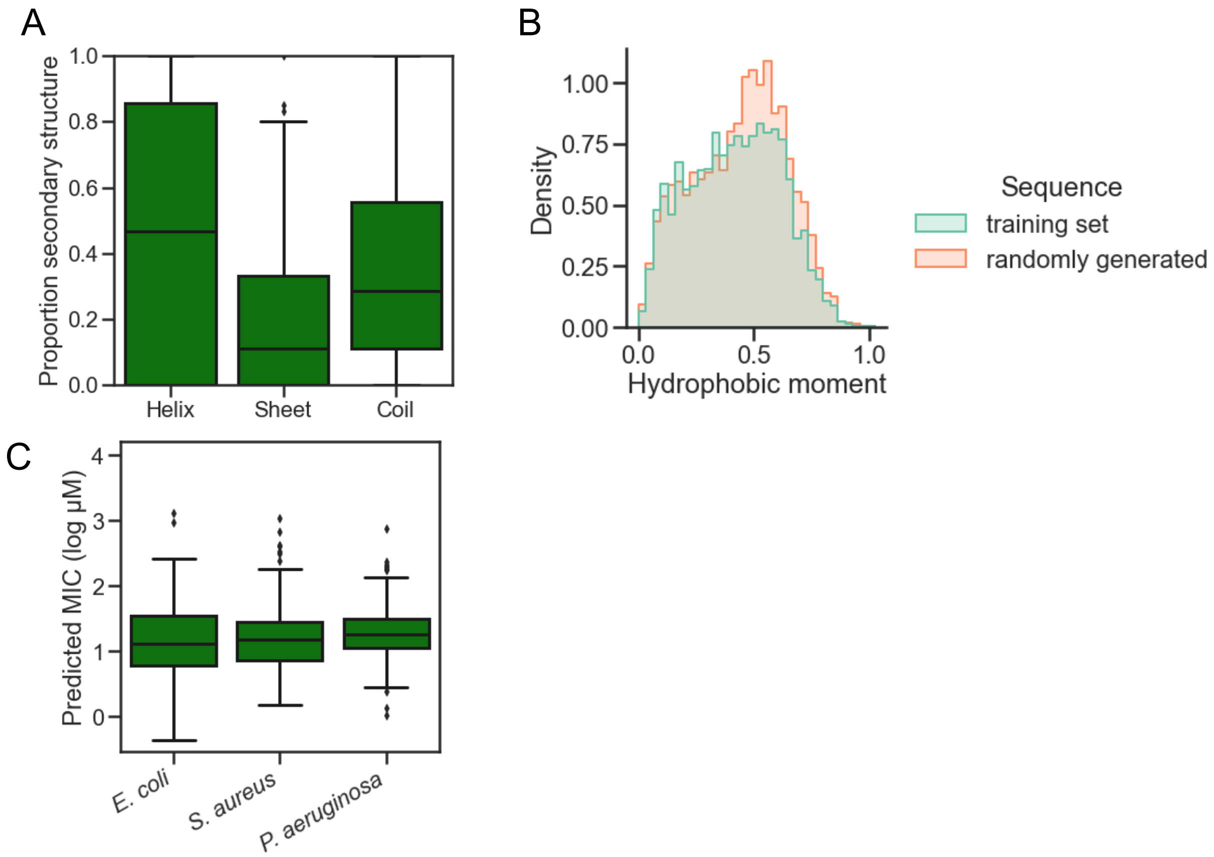

Figure S5. Analysis of randomly generated sequences. Points from latent space were selected at random and decoded to sequences. A) Boxplot of the predicted helix, sheet, and coil percentages calculated via the GOR IV algorithm. B) Density plot of the calculated hydrophobic moments obtained by applying the modlAMP function calculate_moment on the sequences from the randomly generated set and training set. C) A) Predicted MICs for the randomly generated set using the top-performing models for *E. coli*, *S. aureus*, and *P. aeruginosa*.

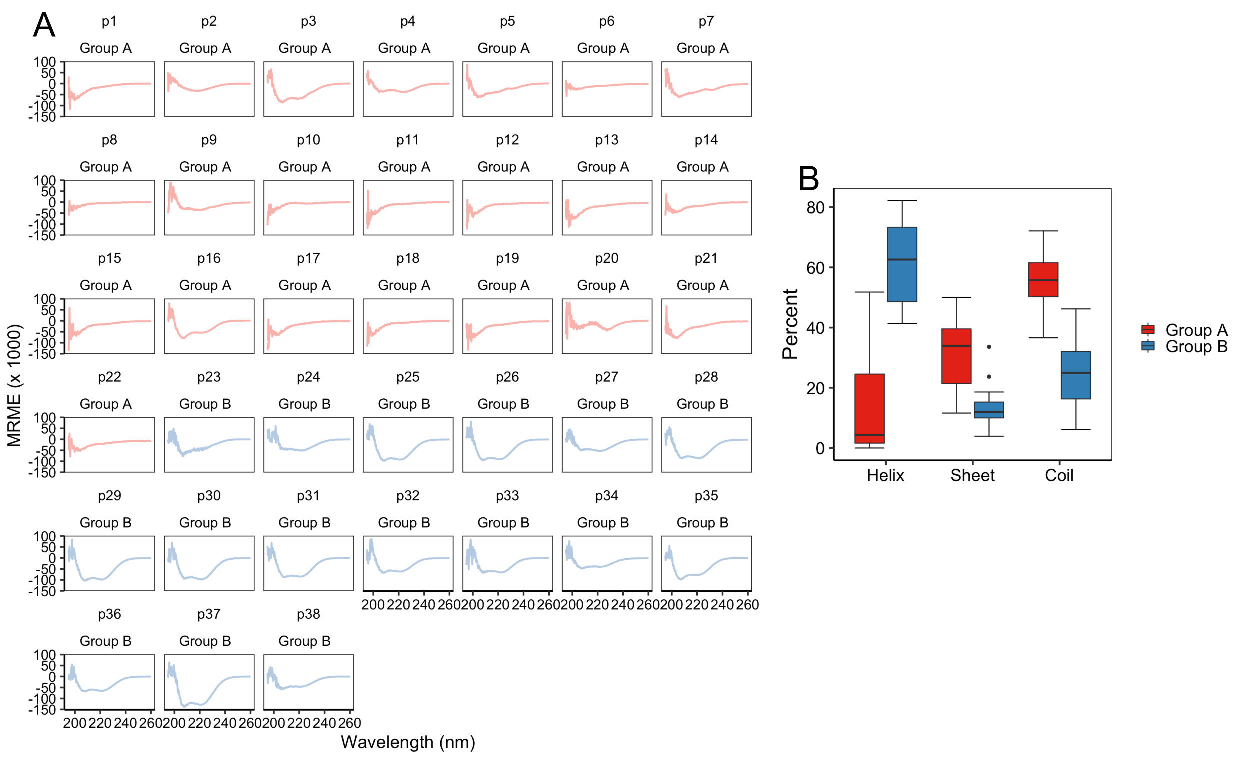

Figure S6. Circular dichroism scans and summary. A) Mean residue molar ellipticity (MRME) plots of peptides from group A and group B in the presence of membrane mimic 60 mM SDS. Each scan was averaged from three scans for each peptide with peptide-free buffer baseline scan subtracted. B) Estimates of secondary structure from circular dichroism data. The Beta Structure Selection CD analysis program was used to obtain helix, antiparallel, parallel, turn, and other. Following summation of antiparallel, parallel, and turn into Sheet, the results were plotted for both groups A and B.

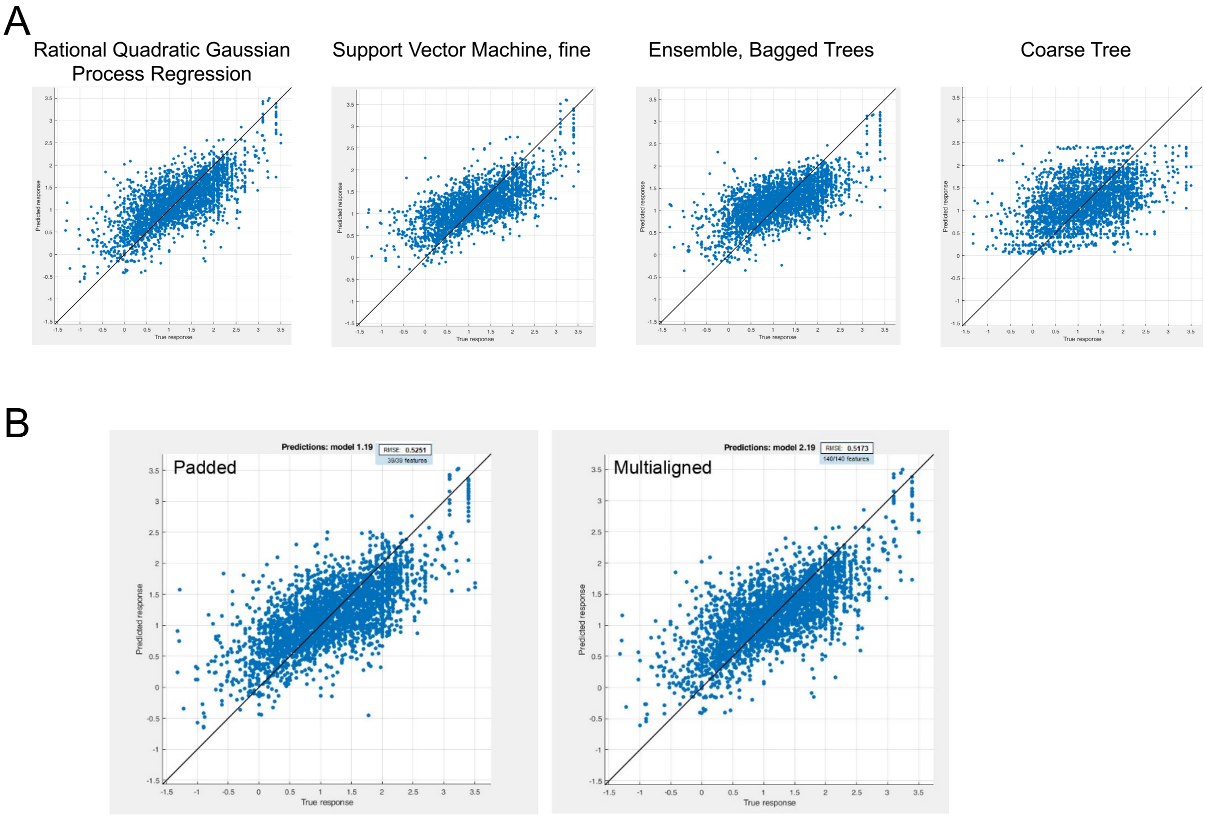

Figure S7. Regression model investigation with MATLAB. A) Representative performance of models trained on *E. coli* dataset from Statistics and Machine Learning Toolbox and Regression Learner app in MATLAB. RMSE values were: Rational Quadratic Gaussian Process Regression: 0.517, Fine Gaussian Support Vector Machine: 0.546, Ensembles of Trees – Bagged: 0.579, and Regression Trees – Coarse: 0.689. B) Comparison of Rational Quadratic Gaussian Process Regression performance using multi-aligned or padded sequences as input.

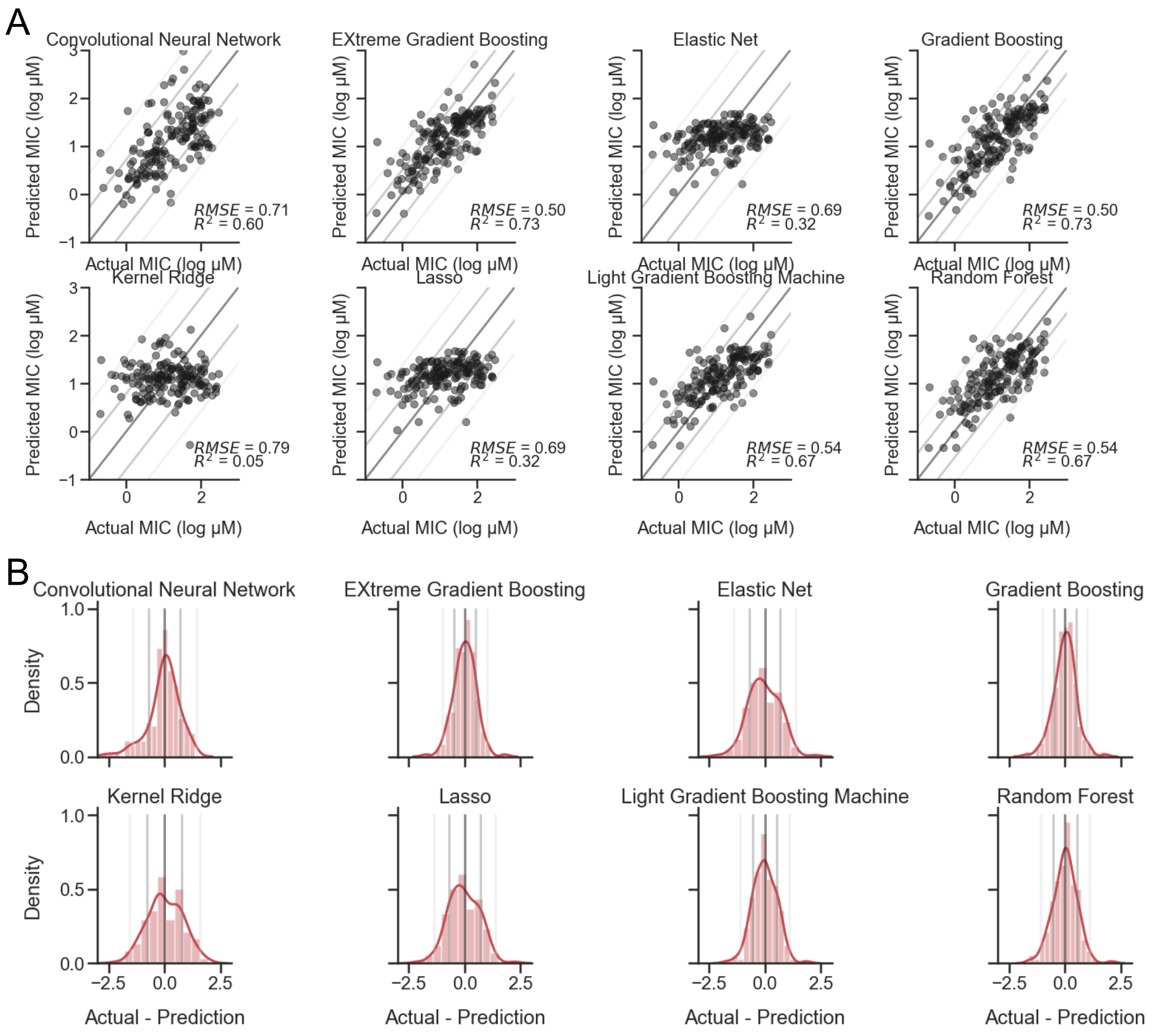

Figure S8. Initial regression model comparison. Eight different regression models for predicting AMP MIC values against *E. coli*. A) Representative scatterplots of Predicted vs. Actual MIC (log µM) of Convolutional Neural Network, Elastic Net, Gradient Boosting, Kernel Ridge, Lasso, and Random Forest, Light Gradient Boosting Machine, and EXtreme Gradient Boosting predictions on holdout test dataset with RMSE and R^2 values displayed. Lines represent standard diagonals, in addition to the diagonal +/- 1 and 2 standard deviations of the points. B) Histograms with overlayed density curves of the Actual - Prediction difference of each model. Lines represent standard diagonals, in addition to the vertical midline +/- 1 and 2 standard deviations of the points.

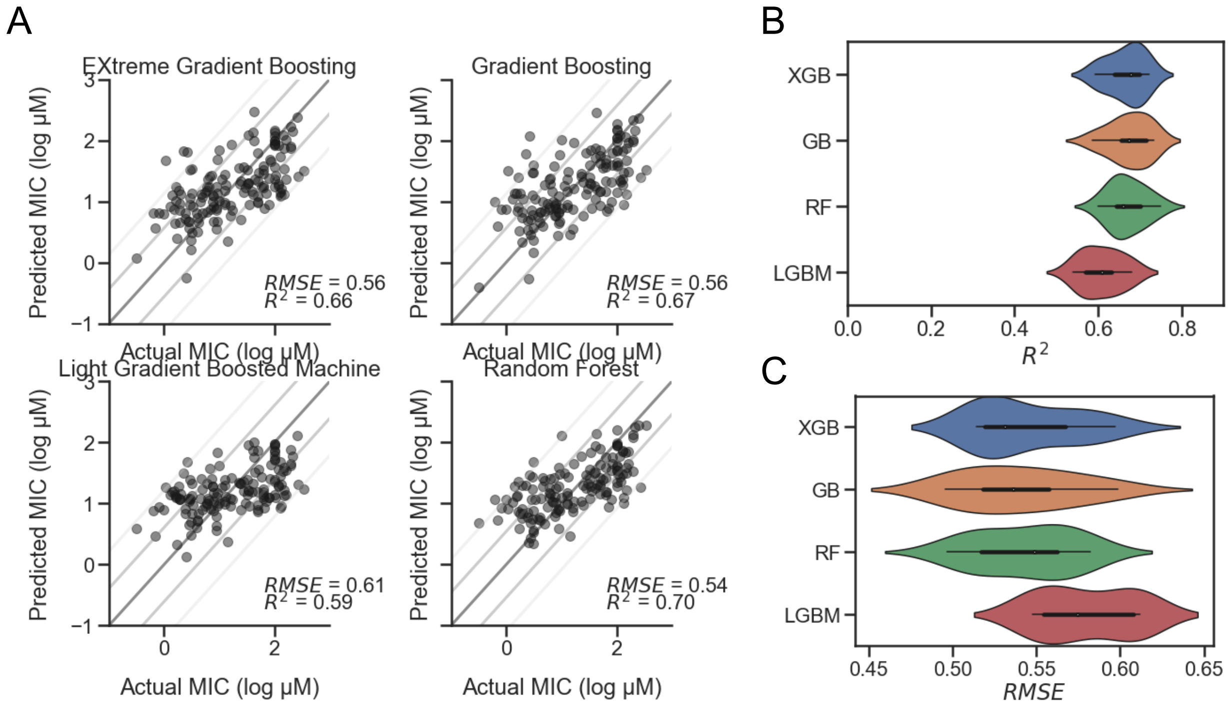

Figure S9. Comparison of MIC prediction models against *S. aureus*. Four regression models for predicting AMP MIC values against *S. aureus*. A) Representative scatterplots of Predicted vs. Actual MIC (log µM) of EXtreme Gradient Boosting (XGB), Gradient Boosting (GB), Light Gradient Boosting Machine (LGBM), and Random Forest (RF) predictions on holdout test dataset with RMSE and R^2 values displayed. Lines represent standard diagonals, in addition to the diagonal +/- 1 and 2 standard deviations of the points. B) Results of cross validation using shuffled split (n=25) shown as a violin plot for each model, sorted from highest to lowest mean R^2 value, with XGB at the top with a median of 0.7. C) Cross validation results for RMSE sorted from lowest to highest mean RMSE value, with XGB at the top with a median of 0.53.

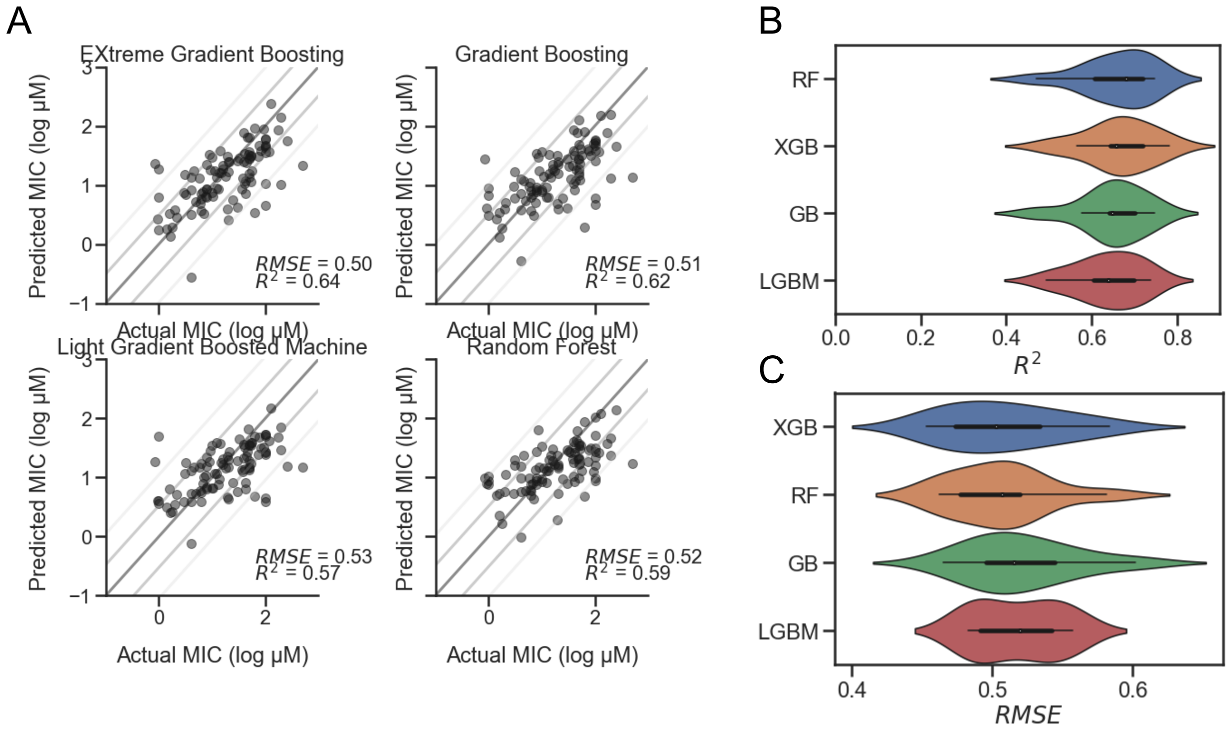

Figure S10. Comparison of MIC prediction models against *P. aeruginosa*. Four regression models for predicting AMP MIC values against *P. aeruginosa*. A) Representative scatterplots of Predicted vs. Actual MIC (log µM) of EXtreme Gradient Boosting (XGB), Gradient Boosting (GB), Light Gradient Boosting Machine (LGBM), and Random Forest (RF) predictions on holdout test dataset with RMSE and R^2 values displayed. Lines represent standard diagonals, in addition to the diagonal +/- 1 and 2 standard deviations of the points. B) Results of cross validation using shuffled split (n=25) shown as a violin plot for each model, sorted from highest to lowest mean R^2 value, with RF at the top with a median of 0.69. C) Cross validation results for RMSE sorted from lowest to highest mean RMSE value, with XGB at the top with a median of 0.5.

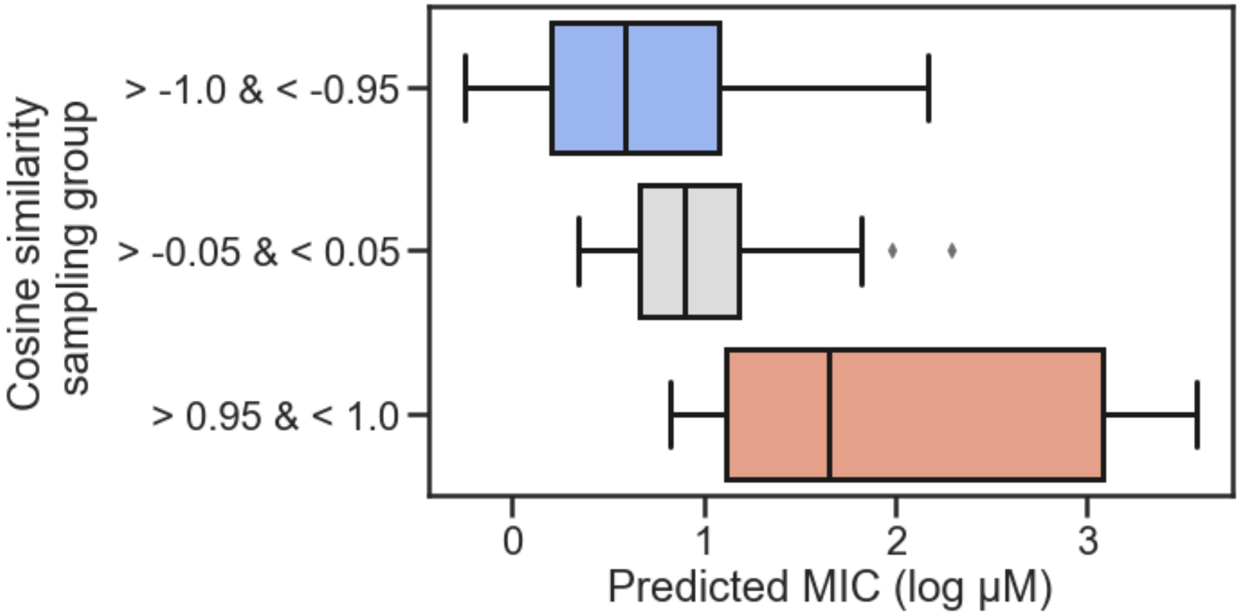

Figure S11. **MIC predictions on cosine similarity sampling groups.** Using the latent vector for VLNENLLA as a reference, vectors for each peptide were sorted by cosine similarity from -1.0 to 1.0 (1.0 being identical to the reference). Peptides were selected at random (n=100) from each of the following groups, filtering on cosine similarity: between -1.0 and -0.95 (> -1.0 & < -0.95), between -0.05 and 0.05 (> -0.05 & < 0.05), and between 0.95 and 1.0 (> 0.95 & < 1.0). New vectors (n=100) were generated near to the selected peptides and decoded to new sequences, to which the *E. coli* MIC prediction model was applied following removal of duplicate sequences. Boxplot summarizes the predicted MICs as a function of cosine similarity group.

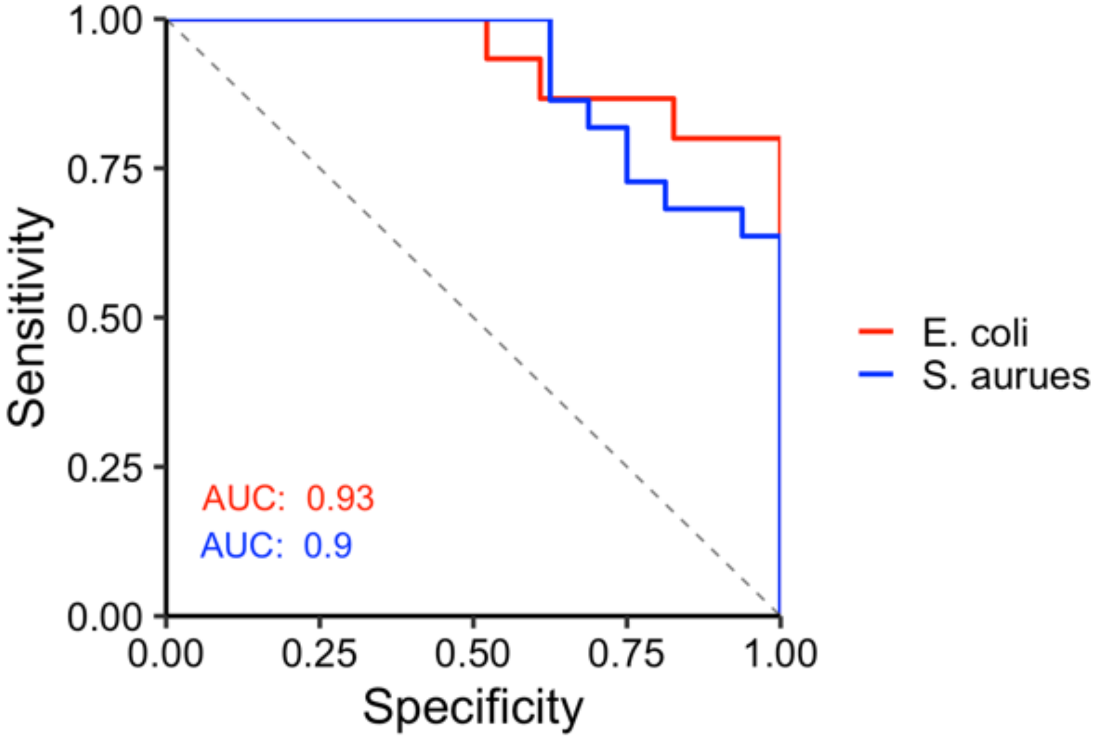

Figure S12. Receiver operating characteristic curve analysis of predicted and experimental MIC results. Receiver operating characteristic (ROC) analysis of predicted and experimental MICs of the 38 synthesized AMPs against *E. coli* and *S. aureus* when categorized into >128 and ≤128 µM yielded area under the curve (AUC) values of 0.93 and 0.9, respectively.
